# Development of Foam Fly Ash Geopolymer with Recycled High-Density Polyethylene (HDPE) Plastics

**DOI:** 10.3390/polym15112413

**Published:** 2023-05-23

**Authors:** Emmanuel M. Atienza, Richard M. De Jesus, Jason Maximino C. Ongpeng

**Affiliations:** Department of Civil Engineering, De La Salle University, Manila 0922, Philippines; richard.dejesus@dlsu.edu.ph

**Keywords:** foam geopolymer, fly ash, recycled HDPE, microstructure, compressive strength, density, thermal conductivity, sustainable alternative material

## Abstract

Adapting sustainable construction, which involves responsible consumption of natural resources and reducing carbon emissions, could be a unified action to address the intensifying effects of global warming and the increasing rate of waste pollution worldwide. Aiming to lessen the emission from the construction and waste sector and eliminate plastics in the open environment, a foam fly ash geopolymer with recycled High-Density Polyethylene (HDPE) plastics was developed in this study. The effects of the increasing percentages of HDPE on the thermo-physicomechanical properties of foam geopolymer were investigated. The samples’ measured density, compressive strength, and thermal conductivity at 0.25% and 0.50% HDPE content was 1593.96 kg/m^3^ and 1479.06 kg/m^3^, 12.67 MPa and 7.89 MPa, and 0.352 W/mK and 0.373 W/mK, respectively. Obtained results are comparable to structural and insulating lightweight concretes with a density of less than 1600 kg/m^3^, compressive strength of greater than 3.5 MPa, and thermal conductivity of less than 0.75 W/mK. Thus, this research concluded that the developed foam geopolymers from recycled HDPE plastics could be a sustainable alternative material and be optimized in the building and construction industry.

## 1. Introduction

One major contributor to the increasing greenhouse gases (GHG) in the atmosphere is the building and construction industry, which is accountable for approximately 36% and 37% of global energy use and energy-related carbon dioxide (CO_2_) emissions [1]. About 82–87% of these emissions came from construction materials [2]. One is cement, which comprises approximately 90% of a building’s structure for its foundations, structural components, and architectural applications [3]. Recent studies show that the cement industry produces over 7% of annual anthropogenic GHG and approximately 8% of global CO_2_ emissions [4]. As the industry’s most used construction material, cement production is expected to expand due to the constant demand for buildings and infrastructures to sustain global population growth and socio-economic developments [5].

Another source of GHG emissions is the increasing volume of plastic waste worldwide. The problem of plastic pollution and climate change have been addressed separately; however, there is a direct relationship between the two [6]. The world produces around 100 million tons of plastic products annually [7]; however, only 9% is recycled, 12% goes to incineration plants, and 79% goes to landfills and the environment [8]. Based on the study of Zheng and Suh [9], every stage of the plastic life cycle generates CO_2_, which includes the disposal of plastic waste in landfills, dumpsites, and oceans. The total GHG from plastic waste was forecasted to further increase by 2050 [10] as the demand for plastic production increases alongside global socio-economic growth.

In response to the worsening global issue of climate change, the construction industry must adopt sustainable building technologies and use energy-efficient, environment-compatible, and sustainable construction materials [11]. This includes reducing cement-based products and reusing existing or waste materials to produce more sustainable alternatives [12]. One material being developed aligned with this objective are geopolymers. Unlike other concrete products, geopolymer is made of a binding system that uses aluminosilicate-rich materials activated by an alkaline solution resulting in a unique reaction known as geopolymerization. The synthesis of geopolymers uses industrial wastes such as fly ash, furnace slag, or clay, making it a more economical construction material. Even without cement in its system, geopolymer can exhibit excellent strength properties while emitting lower amounts of CO_2_ emission [13].

The research development in geopolymers has continued over the past decades to improve its performance and applications in the construction industry. One example is foam geopolymer, a lightweight material with excellent thermal and insulating properties. Like foam concrete, typically made of foam and cement paste or mortar [14], foam geopolymer has a network of pores within its hardened system induced by synthetic or natural foaming agents. Aside from creating pores, the foaming agent plays a vital role as it can control the density of foam geopolymer, usually 200 to 1200 kg/m^3^, while having good strength properties [15]. Given their lightweight and insulating performance, foam geopolymers have the potential as an alternative material to the traditional cement-based products used in construction.

Another timely criterion in achieving a sustainable industry is using waste materials in producing building products such as steel, bricks, and concrete. One of these scrap materials used in the construction sector is High-Density Polyethylene (HDPE), a type of polymer differentiated by its low density, good impact, and chemical resistance properties compared with other recycled polymers such as Polyethylene Terephthalate (PET) and polystyrene (PS). As a thermoplastic, HDPE can be formed into any shape or size without affecting its mechanical properties; thus, HDPE is commonly used as reinforcement, fiber, or filler materials in various concrete products, resulting in notable effects and benefits [16]. In the study of Tamrin et al. [17], the concretes produced with recycled HDPE plastics in different shapes and sizes obtained higher compressive strengths than the targeted and ordinary concretes. On the other hand, the mechanical and serviceability properties of concretes having 0.40%, 0.75%, and 1.25% recycled HDPE plastic fibers have been investigated by Pešić et al. [18], and they concluded that HDPE fibers could be utilized in producing new construction material with notable environmental benefits, as reusing plastics alone could significantly reduce GHG emissions [19].

Various studies have been developed to optimize geopolymers with recycled polymers as additives or fiber reinforcements. In the study of Lazorenko et al. [20], waste PET particles were introduced to geopolymer mortars, resulting in acceptable workability and flexural strength. On the other hand, Kunthawatwong et al. [21] reused polyvinyl chloride (PVC) plastic waste as lightweight aggregates in producing geopolymer mortar. The increasing plastic content improved the water absorption and porosity, reducing density and thermal conductivity by 9–17% and 28–44%, respectively. In contrast, 0–1% Polypropylene (PP) fibers increase the thermal conductivity of fly ash-based lightweight geopolymer concrete. Although at 0.5% PP content, the optimal compressive strength was achieved [22].

In this study, a fly ash-based foam geopolymer with recycled HDPE was developed, aiming to lessen plastic waste and reduce cement use in the building and construction industry. The effects of incorporating HDPE on the foam geopolymer’s system, specifically on its thermo-physicomechanical properties—namely, thermal conductivity, pore characteristics, density, and compressive strength—were investigated to determine its potential use as a sustainable alternative to traditional cement-based lightweight and insulating building materials.

## 2. Materials and Methods

### 2.1. Materials

The foam geopolymer of the study is composed of fly ash (FA), sodium silicate (SS), sodium hydroxide (SH), protein-based foaming agent, and HDPE plastics. The FA was sourced from the coal-fired power plants in Calaca, Batangas, and Mauban, Quezon, Philippines; pre-tested and classified as ASTM C618 Class F fly ash with properties provided by Pozzolanic Philippines, Inc., Taguig, Philippines, as shown in Table 1 [23,24]. As can be seen, the fly ash used has a high concentration of Magnesium Oxide (MgO). Recent studies suggest that MgO is an essential constituent of alkali-activated materials (AAM), including geopolymers, as it can significantly influence its physicochemical reactions and microstructural developments. The fly ash used would contribute to achieving dense foam geopolymers as authors also concluded a more porous microstructure after incorporating high amounts of MgO in their studies [25,26].

**Figure 1:**
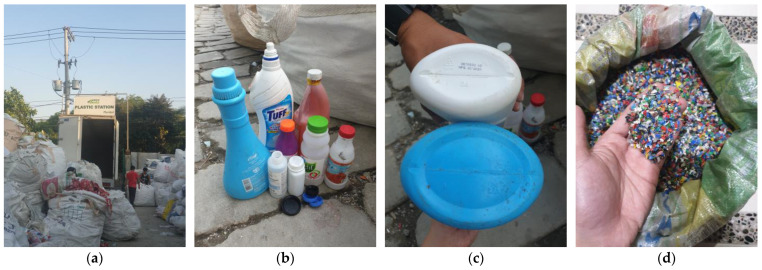
Recycling process: collection (**a**) of HDPE bottles and containers (**b**) with recycling code number “2” (**c**) and shredding (**d**).

The recycled HDPE plastics used are with recycling code number “2”, composed of different colors and sizes of bottle caps, milk jugs, cleaning and laundry detergent containers, shampoo bottles, toothpaste tubes, and cooking oil containers [27]; collected and shredded into 2.5 mm to 3 mm in size by Green Antz Builders, Inc., Plaridel, Bulacan, Philippines, as shown in Figure 1. The sodium silicate contains 15.17% Na_2_O, 33.37% SiO_2_, and 51.46% H_2_O, while caustic soda flakes have 98.20 wt.% purity. The foaming agent is a protein-based, smelly, and color-brown liquid. It comprises 28% animal protein, 2% sodium lauryl polyoxyethylene ether sulfate, 10% sodium dodecyl sulfate, and 60% H_2_O. Distilled water was used in the mixtures to ensure the water was clean and free of any disturbing matter.

### 2.2. Fabrication of Foam Geopolymers

The design mixture used in this study is described in Table 2. A fixed SS:SH mass proportion of 1:2, alkaline activator (AA) to FA percentage ratio of 35%, a fixed dosage of foaming agent at 40% of the total volume, and increasing content of material additives at 0.25% and 0.50% by weight of fly ash were utilized for all geopolymer samples. The amount of HDPE addition was based on the literature. One is the study of Sukmawan and Arifin [28], wherein 0%, 0.25%, 0.50%, 0.75%, 1.00%, and 1.25% Low-Density Polyethylene (LDPE) and PET plastic waste addition were used in their asphalt concrete mixture. The highest stability value was observed with an LDPE and PET variation of 0.50%. In the study of Anum and Job [29], 0%, 0.25%, 0.50%, 0.75%, and 1.0% pulverized HDPE waste were added to their modified concrete mixtures. Results indicate adverse effects on the concrete properties with HDPE content beyond 0.5%. On the other hand, Akter and Raja [30] developed asphalt concrete with 0.25%, 0.50%, 0.75%, and 1.0% shredded expanded polystyrene (EPS) waste and concluded that improved results were obtained at 0.5% addition compared with higher percentage addition. Similar to the study of Sjah et al. [31], by adding 0.5% crushed PP waste, significant improvement was observed in the mechanical properties of concrete samples compared with higher PP content of 0.70%, 1.0%, 2.0%, and 3%.

**Figure 2:**
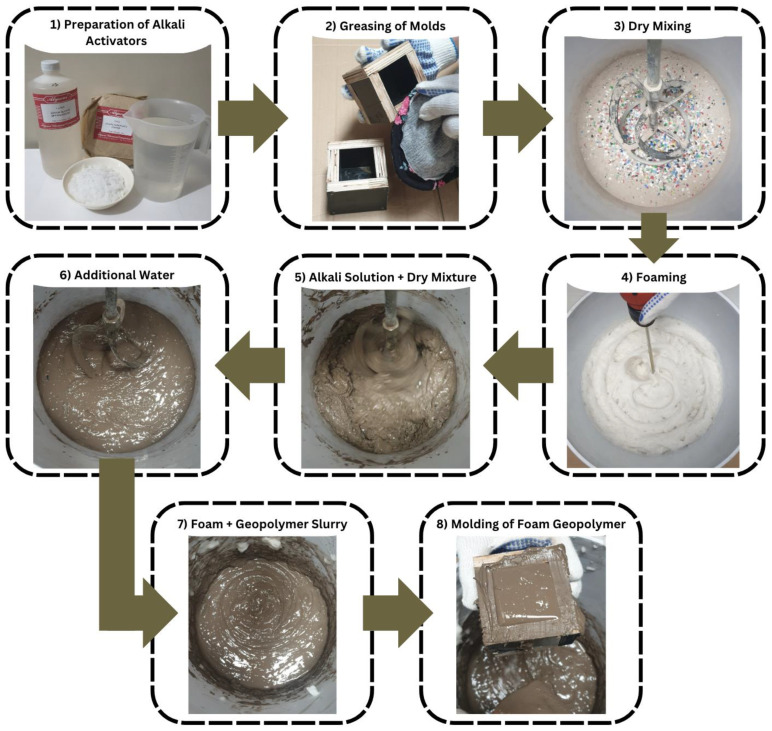
Procedures for foam geopolymer fabrication.

The study used molds with an inside dimension of 50 × 50 × 50 mm, made of ½” phenolic board, and sealed using silicone sealant to avoid or minimize water loss during the casting and hardening of the samples. The step-by-step procedures are illustrated in Figure 2 for fabricating the foam geopolymers.

1. Preparation of the alkaline solution—a mixture of sodium silicate and sodium hydroxide was prepared twenty-four (24) h before use to complete the chemical reaction. The molarity of the sodium hydroxide used in the mixtures is 10M which is performed by dissolving 40 g of caustic soda flakes in 70 mL of distilled water, then adjusting its volume to 100 mL by adding more water. An extra amount of water was added to achieve a sodium silicate solution with 72.25% H_2_O and 27.75% Na_2_O + SiO_2_ and a water content to sum of geopolymer solids (W/GPS) ratio of 0.22.
2. Before the actual mixture, the molds were prepared, and an ample amount of oil was applied throughout the inner surfaces.
3. Dry mixing—using a portable electric concrete mixer, the fly ash and shredded HDPE plastics were mixed slowly until homogeneity was achieved.
4. Foaming—on a separate bucket, foams were created by mixing the foaming agent and water solution at a very high-speed rate using an electric mixer.
5. The prepared alkaline solution was added to the dry mixture and mixed at a speed of 140 rpm for 5 min.
6. The additional water was poured and mixed for another 5 min until a gel-like surface was observed. (Pauses on mixing are necessary to center the slurry into the bucket for a more consistent mixing.)
7. The prepared foams were gradually added at a low speed for 2 min until they spread uniformly into the geopolymer slurry.
8. The fresh geopolymer slurry was placed and compacted in the molds momentarily to avoid flash setting—rapid hardening or setting time after adding an alkali solution.

The molded foam geopolymers were sealed with plastic films to prevent moisture loss. After 24 h of hardening, the samples were carefully de-molded and stored at room temperature of 23 ± 5 °C for 7, 14, and 28 days—the allotted curing period to achieve the desired or maximum strength for the study.

### 2.3. Test Procedures

#### 2.3.1. Scanning Electron Microscopy with Energy Dispersive Spectroscopy (SEM-EDS)

The high magnification imaging of the foam geopolymer samples having 0.00%, 0.25%, and 0.50% HDPE plastics was conducted using JSM IT500HR/LA SSchottky tip Field Emission SEM equipment at Central Instrumentation Facility of De La Salle University Laguna Campus, Biñan, Laguna, Philippines. The apparatus also has Energy Dispersive Spectroscopy (EDS) detectors for elemental identification and analysis. To fit in the SEM chamber, the sizes of the 50 mm cubic samples were reduced to ≤5 mm × 5 mm × 5 mm using a circular saw equipped with a 4″ diameter × 1 mm ultra-thin diamond cutter blade. A lubricant was constantly applied during cutting to reduce friction and avoid damaging the samples. Finally, the cut samples were covered with sputter coating before the SEM imaging to protect the material and the device. The images were taken at the center part of the samples at a magnification of 2000×.

#### 2.3.2. Density Test

The geometric method was applied to determine the density of the samples before conducting the compressive strength test. The weight of each specimen was measured using a digital weighing scale, while the dimensions were measured using a vernier caliper. The arithmetic means of the computed volumetric weights (mass divided by the volume or kg/m^3^) were determined to get the mean density of foam geopolymer with 0.00%, 0.25%, and 0.50%.

#### 2.3.3. Compressive Strength Test

The compressive strength test was conducted using a TBT TYA-2000 KN digital compression machine following the ASTM C109/C109M-08 [32] procedures at A’s Geotechnical, Inc., Dagupan City, Pangasinan, Philippines. The cube samples with 50 × 50 × 50 mm dimensions were tested after 7, 14, and 28 days of curing. After five replicates were tested, the data were averaged.

#### 2.3.4. Thermal Conductivity Test

The thermal conductivity of the foam geopolymer with 0.00%, 0.25%, and 0.50% was determined using a KD2 Pro thermal properties meter at Geotechnics Philippines, Inc., Quezon City, Metro Manila, Philippines. Aligned with ASTM D5334 [33], KD2 Pro has a 2.4 mm-diameter × 100 mm-long needle, thermal conductivity reading range of 0.10–4.00 W/mK, and a ±10% accuracy. The thermal conductivity was measured by the following method: (1) cutting the prepared sample into halves and stacking them to achieve dimensions of 100 mm × 50 mm × 25 mm, (2) drilling a hole throughout the sample using a 4 mm drill bit, enough to cover the entire needle, (3) inserting the needle into the sample, (4) applying thermal compound to the tip hole and other gaps to eliminate entrapped air and enclose the sample, and (5) performing a thermal conductivity reading within 10 min.

## 3. Results and Discussions

### 3.1. SEM-EDS Analyses

**Figure 3:**
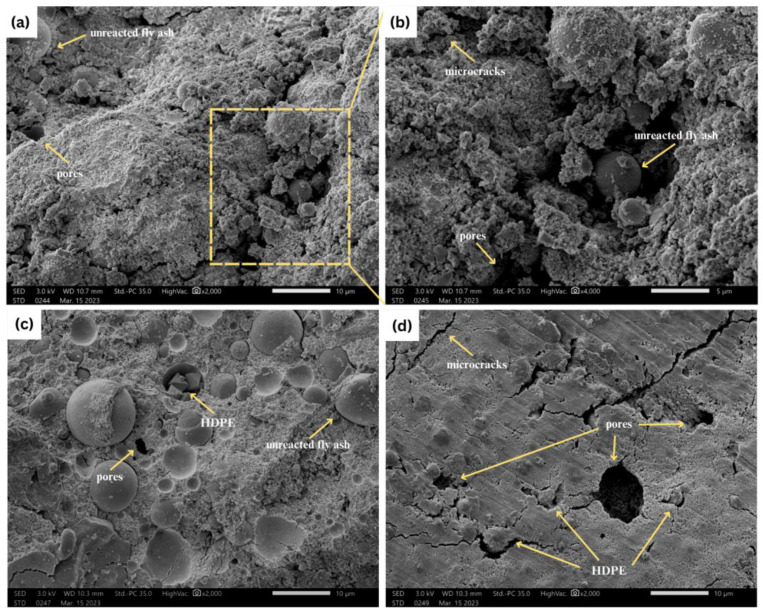
SEM images of the samples with (**a**) 0.00%, (**b**) 0.00% at 4000×, (**c**) 0.25%, and (**d**) 0.50% HDPE.

The SEM imaging result shows the surface morphology of the foam geopolymer sample with 0.00% HDPE at 2000× magnification, 0.00% HDPE at 4000× magnification, 0.25%, and 0.50% HDPE at 2000× magnification, as shown in Figure 3. Unreacted fly ash or the incomplete reaction of fly ash with alkali activators can be observed more dominantly on the foam geopolymers with 0.00% and 0.25% HDPE plastics, possibly caused by the curing method applied to the samples. It is evident from the literature that by increasing the curing temperature, an acceleration in the dissolution, polymerization, and reprecipitation within the geopolymerisation process can be observed, which results in geopolymers’ improved performances. In the study of Promentilla and Ngo [34], denser structures and lesser unreacted fly ash can be seen in the foam geopolymers cured under higher temperatures of up to 75 °C. The sample with 0.50% HDPE has the least unreacted fly ash; however, a significant number of microcracks can be found in its structure. In Figure 3c,d, HDPE plastics can be found within some air voids or microcracks. According to Jhatial et al. [35], this occurrence is because of the hydrophobic nature of polymers which keep water and creates gaps that easily separate them from the matrix, especially in the hydration process of cement-based concretes and the gelation phase of geopolymer mixtures.

Thus, SEM findings show that the air voids and microcracks may also increase with increased HDPE content, resulting in lower densities and weaker strengths. It is an important indicator that could be used as a reference and qualitative validation for further discussions about the resulting thermo-physicomechanical properties of the foam geopolymers after undergoing density, compressive strength, and thermal conductivity tests.

The fundamentals of the geopolymerization process are (1) the dissolution of the aluminosilicate-rich material in the highly concentrated alkali solution, (2) the gelation phase, and (3) the hardening phase, forming a three-dimensional silicoaluminate structure that forms a geopolymer, mainly composing of Al, Si, and O, with a small amount of Na and K [36,37]. To confirm the presence of these essential elements within the foam geopolymers’ system, the images captured by SEM were also subjected to EDS analysis. According to Moro et al. [38], SEM-EDS is one practical approach for microstructural characterization and elemental identification and quantification of a geopolymer composite.

**Figure 4:**
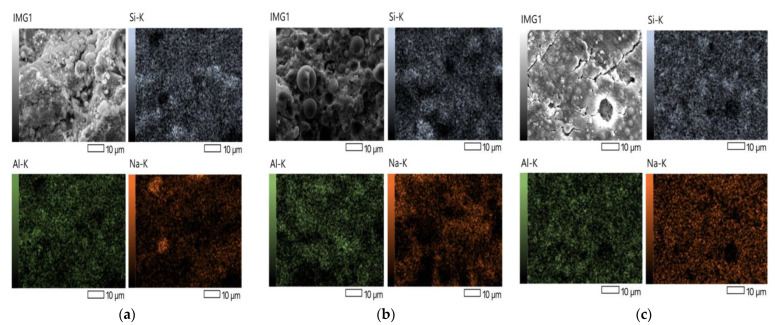
EDS analysis of foam geopolymer with 0.00% (**a**), 0.25% (**b**), and 0.50% (**c**) HDPE.

Thus, EDS shows a high composition of Si, Al, and O, and traces of Na, Ca, Mg, Ti, and C, tallying to a Si/Al ratio of 2.6, 2.6, and 2.7 for foam geopolymers with 0.00%, 0.25%, and 0.50% HDPE, respectively, as shown in Table 3. Based on the result, the Si/Al ratio of the foam geopolymers satisfies the typical Si/Al ratio of geopolymers at 1–3 [39] and the Si/Al ratio of low carbon emitting cement or concretes at 2:1 [40]. On the other hand, Figure 4a–c show the uniform distribution of the Si, Al, and Na within the captured area of foam geopolymers with 0.00%, 0.25%, and 0.50% HDPE addition, respectively.

Aside from the curing conditions, the Si/Al molar ratio can also affect the geopolymerization process [41,42]. Though the synthesis of the foam geopolymer in this study uses a fixed amount of AA:FA and SS:SH ratios, the computed value of the Si/Al ratio, which is related to the volume of alkali activators used, could also be responsible for the presence of unreacted fly ash seen in the SEM images of the samples. In the study of Al-Rkaby et al. [43], SEM-EDS also shows partially reacted FA particles throughout their geopolymer gel, caused by insufficient activators to decompose silica and alumina with FA. Thus, according to the literature, increasing the SS:SH ratio increases the silica content and Si/Al ratio, resulting in a higher degree of geopolymerization that causes fewer unreacted FA spheres, a denser structure, and higher strengths of a geopolymer matrix [44,45].

Meanwhile, FA also plays a vital role in achieving a porous structure of foam geopolymers [46]. The fine-size particles of FA interact with the bubbles, providing a coating that causes solid-like walls, which later become pores after evaporation and hardening of the geopolymer matrix, as shown more noticeably in Figure 3c. This observation is evident in recent studies exploring the effects of different dosages of FA replacement or additives to foam concrete mixtures. In the study of Zhang et al. [47], the 10–20% addition of FA improved the pore structure of foam concrete and created a smaller size of pores. By increasing it to 40%, the highest porosity was obtained. On the other hand, Tao and Dong [48] found that increasing FA proportions for cement replacement increases porosity, reducing the compressive and tensile strength of foam concretes.

### 3.2. Density

**Figure 5:**
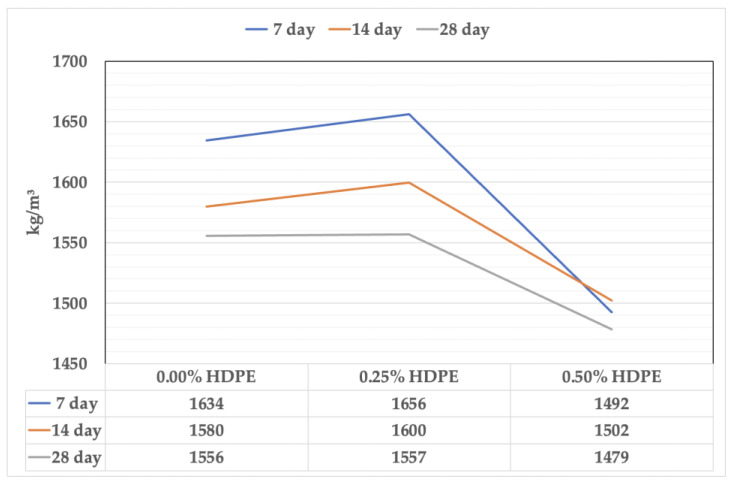
The density of the foam geopolymers.

The effect of HDPE addition on the density of the foam fly ash geopolymer is described in Figure 5. A minimal increase in the samples’ density was observed after adding 0.25% and remarkably decreased after having a full HDPE content of 0.50%. It is noticeable that the density of the samples lowers after 14- and 28-day curing periods, wherein the lowest densities were recorded from 28-day-old samples, which are foam geopolymer with 0.50% HDPE at 1479 kg/m^3^ and 0.25% HDPE at 1557 kg/m^3^—5% lower and 1% higher than the reference mix at 1556 kg/m^3^, respectively.

The outliers were detected from the dataset and interpreted using box plot analysis to observe a more consistent calculation of the foam geopolymers’ mean densities. This range of values is associated with the flash setting or rapid hardening of the geopolymer slurry. The unanticipated occurrence causes inconsistencies with the compaction, especially in the latter part of the molding process. To minimize the error, it is crucial to follow the procedure described in Section 2.2; specifically, the molds must be prepared before the mix to ensure the on-time and momentarily casting of slurry into the molds. Prolonging the setting time is still a challenge as it dramatically affects the workability of some geopolymers considering the transportation, placing, and compaction in small- to large-scale projects [49].

**Figure 6:**
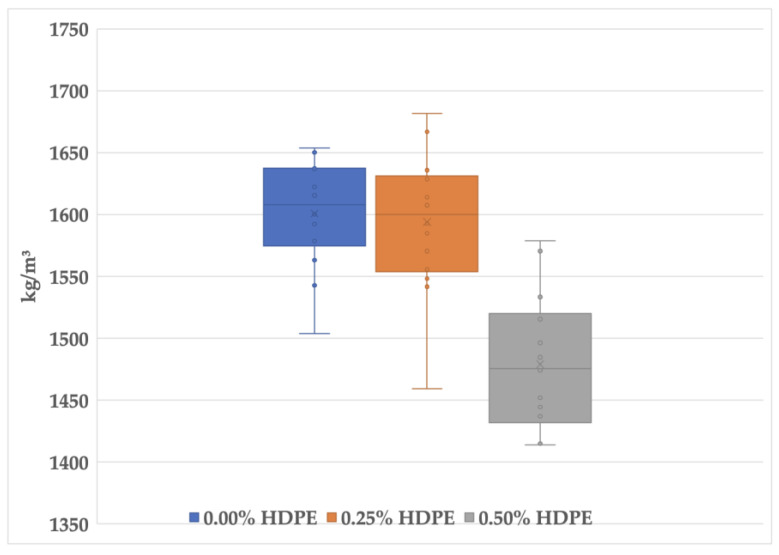
Box plot of the foam geopolymers’ density.

In addressing the inconsistencies, the detected outliers were removed from the dataset to more accurately analyze the effects of the increasing percentages of HDPE on the densities of the foam geopolymer samples. As shown in Figure 6, the computed mean values (marked “×”) are 1601 kg/m^3^, 1594 kg/m^3^, and 1479 kg/m^3^ for foam geopolymers with 0.00%, 0.25%, and 0.50% HDPE plastics, respectively. The result indicates that increasing the HDPE content lowers the foam fly ash geopolymer density.

As discussed in the microstructure analysis of the foam geopolymer samples, HDPE is hydrophobic. Due to its immiscibility, adding such polymer to concrete mixtures increases the air content, affecting its physical properties [50]. Moreover, some reported cases are caused by moisture loss during the hydration and curing processes that affect the compactness of the concrete mixture. In the study of Tamrin and Nurdiana [51], adding 2.5%, 5%, 10%, and 20% (by weight of cement) of 0.5 mm-thick lamellar HDPE decreases the unit weight of medium concretes. Similarly, the density of a concrete with 0% to 15% recycled HDPE as fine aggregate lowers from 2165 kg/m^3^ to 1825 kg/m^3^, respectively [52].

### 3.3. Compressive Strength

**Figure 7:**
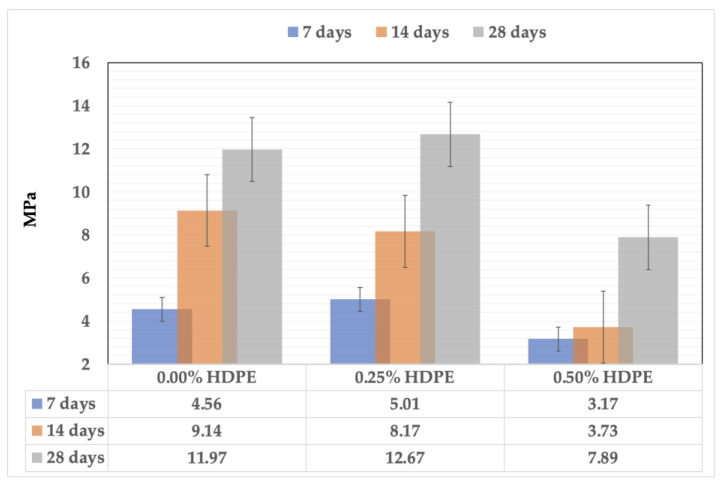
The compressive strength of the foam geopolymers.

The compressive strength of the foam geopolymer samples was measured complying with ASTM C109/ C109M, as there is no separate standard for geopolymers to-date. Similar to the concrete standard EN 12390–3 adapted by Kozub et al. [53] in determining the compressive strength of foam geopolymers with glass wool waste, 50 mm cubic samples were subjected to a gradual load rate of 0.04 MPa/s, and the maximum load at failure were recorded accordingly. The computed average of five replicates per design mix at 0.00%, 0.25%, and 0.50% HDPE cured within 7, 14, and 28 days are tabulated and interpreted in Figure 7.

The samples’ strength was developed throughout the curing period, and the maximum values were recorded from their 28th day. The 7-day compressive strength of the samples without HDPE content gently increases at 9% and decreases at 30% with the 0.25% and 0.50% HDPE addition, respectively. The same trend exists with the 28-day strength values of the samples, which slightly rises at 6% and notably lowers at 34% when the HDPE addition is at 0.25% and 0.50%, respectively. The highest compressive strength is 12.67 MPa from the foam geopolymer with 0.25% HDPE content, followed by the reference mix with 11.97 MPa, and the foam geopolymer with 0.50% HDPE at 7.89 MPa gave the lowest. Thus, the results show that the greater the HDPE plastics incorporated, the lower the compressive strength obtained.

**Figure 8:**
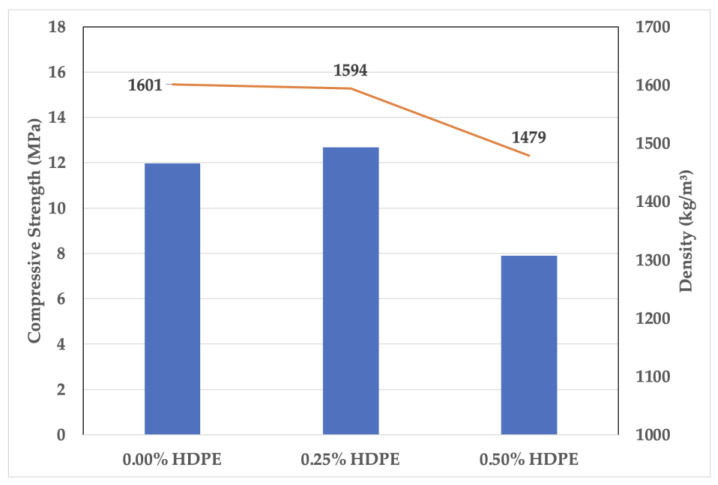
Compressive strength vs. density of the foam geopolymers.

Foam geopolymer has a porous structure. Aside from the pores created by the foams incorporated into the mixture, SEM images show air voids and microcracks, more significantly on the geopolymers that contain 0.50% HDPE plastics. This connotates that the increase in HDPE content increases the air content volume, lowering the density and, consequently, the compressive strength, as shown in Figure 8.

Moreover, Sajan et al. [54] concluded that a more extended curing period of fly ash-based geopolymers may result in lower strengths because of moisture loss. Likewise, in this study, curing the foam geopolymer samples frees the water entrapped by HDPE within the matrix, which causes gaps and microcracks in its surrounding area. However, according to Kioupis et al. [55], a compressive strength of 2 MPa and above can be achieved despite the porous structure of foam geopolymers, which is clear from a foam geopolymer with a maximum strength of 11.97 MPa that has been developed in this study.

### 3.4. Thermal Conductivity

Thermal conductivity is the amount of heat that passes through the wall. The lower the thermal conductivity for insulating material, the better, as lesser heat energy is conducted. Studies also show that minimizing heat gain from walls can reduce energy consumption for cooling systems [56], resulting in energy savings and decreased energy-related carbon emissions [57].

The measurement of the thermal conductivities of the foam geopolymers follows ASTM D5334 using a KD2 Pro meter. After subjecting each sample to an average temperature of 25.52 °C, the 0.25% HDPE addition lowered the thermal conductivity by almost 6%, while the 0.50% HDPE addition resulted in the same value as the reference mix. According to Fatimah et al. [58], foam geopolymer’s thermal properties depend on its porosity structure. As the volume of pores increases, the density will decrease, and lower thermal conductivity may be obtained. In the study of Badache et al. [59], the thermal conductivity of lightweight composite mortar lowers as the HDPE sand content increases due to the significant volume of pores and low thermal conductivity of HDPE sand at 0.4 W/mK compared to the conventional sand. Similar observations were made by Aattache et al. [60], wherein the thermal conductivity improved as the density of the mortar decreased with the increased HDPE for cement replacement.

**Figure 9:**
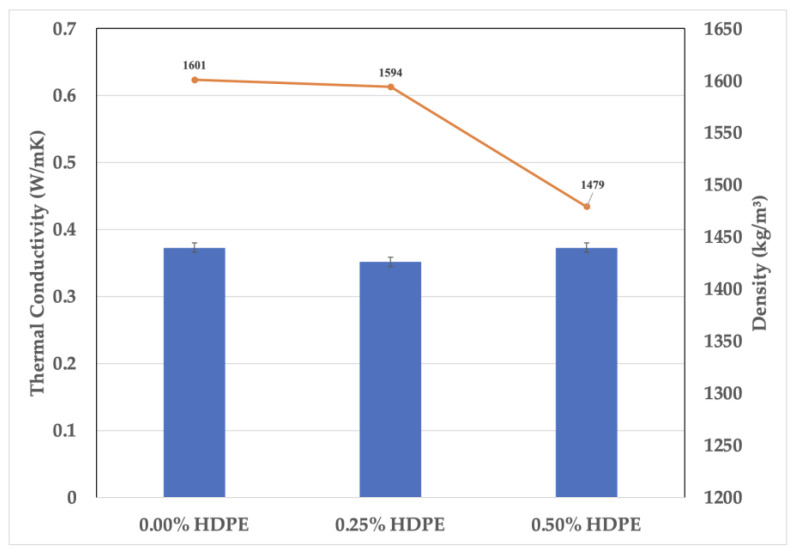
Thermal conductivity vs. density of the foam geopolymers.

As shown in Figure 9, the foam geopolymer with 0.50% HDPE had the lowest density at 1479 kg/m^3^; however, its thermal conductivity is the same as the reference mix, which has a higher density of 1601 kg/m^3^. A stabilized thermal conductivity of foam geopolymer samples was observed after the HDPE addition.

Aside from the physical properties, thermal conductivity depends also on heat transfer conditions [61]. However, the absence of standards for geopolymer lets authors adopt different methodologies and test procedures. In the study of Agustini et al. [62], the measurement of the thermal conductivity was performed by subjecting their samples to increasing temperatures of 50 °C, 100 °C, 150 °C, and 200 °C, complying with ASTM C177. As a result, their foam geopolymer with 0.25% and 0.50% polypropylene had the lowest thermal conductivity of 0.212 W/mK and 0.470 W/mK at 200 °C, which is 72% and 6% lower when at 50 °C, having 0.766 W/mK and 0.499 W/mK, respectively. Similar methodology and results were used by Aattache et al. [60], wherein the cement mortar with 2%, 4%, and 6% HDPE replacements was subjected to 20 °C, 140 °C, 250 °C, and 350 °C, aligned with ASTM D5930.

A substantial effect on the thermal properties of concretes was also observed with higher percentages of plastic addition or replacement. At an average temperature of 25 °C, it was found that the thermal conductivity of common polymers such as HDPE, LDPE, and PP is 0.43 W/mK, 0.35 W/mK, and 0.23 W/mK, respectively, which is three to thirteen times lower than the typical thermal conductivity of ordinary concretes ranging from 1.34–2.92 W/mK [63]. In the study of Kioupis et al. [64], 0.5%, 1%, 1.5%, 2%, 2.5%, and 3% of EPS plastics were added, resulting in a significant reduction of up to 40% (at maximum EPS content of 3%) in the thermal conductivity of the fly ash-based geopolymer. On the other hand, Bahij et al. [65] achieved 6.6%, 12.1%, and 15.5% reductions with 0.25%, 0.50%, and 0.75% non-woven PET addition to the concrete mixtures.

### 3.5. Summary of Test Results

The need for comprehensive design terminologies and methodologies is one factor in the absence of general standards for geopolymers [66]. Thus, the literature and recent studies became the scientific foundation for developing foam geopolymers in this study. According to Ming et al. [15], foam geopolymers typically have a 200–1200 kg/m^3^ density, compressive strength below 10 MPa, and excellent insulation properties. However, the foam fly ash geopolymers with 0.00%, 0.25%, and 0.50% HDPE content have a density, compressive strength, and thermal conductivity ranging from 1601–1479 kg/m^3^, 11.97–7.89 MPa, 0.373–0.352 W/mK, respectively.

Nevertheless, as the ultimate goal is to develop an alternative to lightweight and traditional cement-based insulating construction materials, the measured thermo-physicomechanical properties of the foam geopolymers with HDPE plastics were also compared to the properties of lightweight concrete recommended by RILEM [67]. The properties of foam geopolymers with 0.00%, 0.25%, and 0.50% HDPE plastics all satisfy the required density, compressive strength, and thermal conductivity of a structural and insulating lightweight concrete of <1600 kg/m^3^, >3.50 MPa, and <0.75 W/mK, respectively.

## 4. Conclusions and Recommendations

The potential use and capability of foam fly ash geopolymer with recycled HDPE plastics as an alternative lightweight and insulating building material was determined based on the laboratory tests conducted in the study. The effects of 0.25% and 0.50% HDPE addition to the thermo-physicomechanical performance of foam geopolymer were investigated through SEM-EDS, density, compressive strength, and thermal conductivity tests.

The SEM images show the surface morphology of the foam geopolymers before and after adding HDPE plastics. Due to the hydrophobicity and immiscibility of HDPE, water content is entrapped between the polymer and geopolymer matrix during the hydration process and then freed during the curing period, resulting in the formation of air voids and microcracks. This explains the reduction in the density and compressive strength of foam geopolymer by almost 8% and 34% at a maximum HDPE addition of 0.50%. Nonetheless, the foam geopolymer with 0.25% and 0.50% HDPE has a density of 1594 kg/m^3^ and 1479 kg/m^3^, compressive strength of 12.67 MPa and 7.89 MPa, and thermal conductivity of 0.352 W/mK and 0.373 W/mK, respectively, which are comparable with the properties of structural and insulating lightweight concrete. The obtained values indicate that the developed foam geopolymers with recycled HDPE plastics could be one alternative to cement-based products. Optimizing its use could be a sustainable approach to reducing carbon emissions from the construction and waste sector and eliminating plastic pollution across the globe. The result could also be a reference and contribute to future initiatives for geopolymers’ use towards sustainability.

For future studies, authors may consider adjustments to the study’s processes to enhance the overall performance of foam geopolymer. A SS:SH ratio of 1:2 was used for the geopolymer mixtures, resulting in a Si/Al ratio of 2.6. The samples were also cured at room temperature. These two conditions are factors in the presence of unreacted fly ash or incomplete reactions within the geopolymer matrix. Studies suggest that increasing the Si/Al ratio and curing geopolymers under higher temperatures may cause a higher degree of geopolymerization which causes denser structures and improved properties. The rapid hardening of foam fly ash geopolymer with recycled HDPE plastics must also be addressed to improve its workability and application for large-scale projects.

## Figures and Tables

**Table 1:** Chemical composition and properties of Class F fly ash.

Composition and Properties	Content (%)
Silicon Dioxide (SiO_2_)	57.2
Aluminum Trioxide (Al_2_O_3_)	21.8
Ferric Oxide (Fe₂O₃)	4.73
Calcium Oxide (CaO)	6.9
Magnesium Oxide (MgO)	9.9
Loss of Ignition (LOI)	0.6
Insoluble Residue (IR)	55.1
Sulfur Trioxide (SO₃)	1.23
Moisture Content	0.1

**Table 2:** Design of the Experiment.

AA to FA% Ratio	SS: SH Ratio	% of Foaming Agent	% of HDPE Addition
35	1:2	40	0.00
			0.25
			0.50

**Table 3:** Elemental composition of the foam geopolymers by mass %.

HDPE%	C	O	Na	Mg	Al	Si	K	Ca	Ti	Total	Si/Al
0.00%	7.1	52.5	7.7	1.0	7.2	19.1	1.0	1.0	3.4	100	2.6
0.25%	10.0	49.9	8.8	1.1	6.6	17.4	2.0	1.3	2.9	100	2.6
0.50%	8.9	49.8	6.1	1.1	7.5	20.4	1.1	4.7	0.4	100	2.7

## Data Availability

Not applicable.
